# Analysis of Tonguing and Blowing Actions During Clarinet Performance

**DOI:** 10.3389/fpsyg.2018.00617

**Published:** 2018-04-30

**Authors:** Montserrat Pàmies-Vilà, Alex Hofmann, Vasileios Chatziioannou

**Affiliations:** Department of Music Acoustics, University of Music and Performing Arts Vienna, Vienna, Austria

**Keywords:** clarinet, articulation, tongue, single-reed, player-instrument interaction

## Abstract

Articulation on the clarinet is achieved by a combination of precise actions taking place inside the player's mouth. With the aim to analyse the effects of tonguing and blowing actions during playing, several physical variables are measured and parameters related to articulation are studied. Mouth pressure, mouthpiece pressure and reed displacement are recorded in an experiment with clarinet players to evaluate the influence of the player's actions on the selected parameters and on the sound. The results show that different combinations of tongue and blowing actions are used during performance. Portato and legato playing show constant blowing throughout the musical phrase, which varies according to the dynamic level. In portato, short tongue-reed interaction is used homogeneously among players and playing conditions. In staccato playing, where the tongue-reed contact is longer, the mouth pressure is reduced significantly between notes. Such a mouth-pressure decrease might be used to stop the note in slow staccato playing. It is hereby shown that when the note is stopped by the action of the tongue both the attack and release transients are shorter compared to the case where the vibration of the reed is stopped by a decrease of mouth pressure.

## 1. Introduction

For expressive woodwind performance musicians use various articulation techniques to shape the sequences of notes. The large variety of these techniques include different kinds of tongue actions (Krautgartner, 1982). Most cases require direct interaction of the player's tongue with the oscillating reed (Scavone, 1996), involving accurate control of the front portion of the tongue, in order not to change other factors of the embouchure (Teal, 1963). For instance, in tongue-articulated series of notes, woodwind players are taught to maintain the embouchure parameters without stopping the airflow into the mouthpiece (Brymer, 1976).

The air pressure in the player's mouth is the main parameter controlling the dynamic level of the produced sound. Typically in musical acoustics, the pressure in the mouth is considered as a measure of his/her blowing technique, as it is more straightforward to obtain than any other measurement of respiratory actions (Bouhuys, 1968; Fréour and Scavone, 2013). The required blowing pressure depends on the desired dynamics and it is also closely related to other factors, such as the strength of the reed, the register and/or the articulation technique (Fuks and Sundberg, 1999). Furthermore, playing a certain task in a German-system clarinet generally requires higher blowing pressure than an analogous task in a French-system clarinet (Nederveen, 1969).

The articulation techniques can be broadly classified as: *legato* with a steady air support during and between notes and no tongue-reed contact, *portato* with a fast tongue-reed stroke between notes and *staccato* with detached, short notes (Ellsworth, 2014). The articulation controls the duration of the notes and the characteristics of attack and release transients of every note. Other features such as the position of the tongue stroke on the reed or the intensity of the contact do also contribute to control the transients of the sound (Liebman, 1994; Lawson, 1995). In staccato playing, Bonade (1949) explains that the tempo of the performance is related to the length of the notes. Faster tempo involves shorter staccato-articulated notes than slower tempo. The shorter the notes, the longer the tongue remains on the reed, while maintaining the air flow into the instrument, as the tongue acts as a release valve when it loses contact with the reed. The two more common strategies to stop a sound are to dampen the reed vibrations by contacting the reed with the tongue, or to reduce the mouth pressure below the oscillation threshold (Guillemain et al., 2014). Other techniques to stop the sound might involve actions with the lip or with the back of the tongue (as in double-tonguing) to interrupt the airflow into the instrument (Liebman, 1994).

Considering these theoretical explanations by clarinet experts, we are interested in systematically exploring the properties of articulation. To that end, we aim at obtaining characteristic values for a set of measurable parameters related to the blowing and the tonguing actions during note transitions. The relationship between these parameters and the properties of the music (dynamics, tempo) are investigated. With this information, a further insight into articulation techniques is to be provided, as well as the possibility to explain the influence of tonguing and blowing actions on the sound that is finally obtained.

Exploration of tongue and blowing actions during articulation is not straightforward, as they remain out of visual inspection. Previous studies consider the pressures at the player's mouth and inside the instrument's bore (mouthpiece or barrel) together with measurements of the lip force (Guillemain et al., 2010) or tongue-reed contact instants (Li et al., 2016) to analyse the attack and release transients when playing isolated notes. Other studies consider the tongue physiology to describe its motion and the way it contacts the reed during articulation (Anfinson, 1969; Lulich et al., 2017). With the aim to analyse the tongue timing skills and its coordination to the fingers' actions, reed bending measurements have been used on the saxophone (Hofmann and Goebl, 2014) as well as together with finger sensors on the clarinet (Hofmann and Goebl, 2016).

In this paper, we aim at providing a new experimental approach by combining blowing and mouthpiece pressure measurements with reed bending measurements, with the objective to identify the playing parameters present in the coordinated tongue-blowing actions that result in a certain expression or musical effect. To that end, we present the methodology used to measure and extract the parameters related to articulation (section 2) and we provide the results of an empirical analysis of clarinet performance (section 3) in order to describe and discuss the observed characteristics of articulation (section 4).

## 2. Materials and methods

### 2.1. Participants

In the experiment, eleven clarinettists (*N* = 11) were asked to play selected music exercises and excerpts under controlled performance conditions, recorded individually in eleven experimental sessions. All participants were over 18 years old (average of 23 years) and were playing the clarinet as their main daily activity. Two of them were professional clarinet players (with more than 16 years of practice) and 9 of them were advanced students of the University of Music and Performing Arts Vienna (7–14 years of practice). The participants volunteered to take part in the experiment and received a nominal fee.

### 2.2. Experimental set-up

For the current study, the experiments were performed using a German B♭ clarinet and mouthpiece (Thomann GCL-416 Synthetic Line; Maxton NA-1), and a synthetic clarinet reed, whose characteristics are independent of humidity. All participants were asked to use the same instrument, mouthpiece and reed, under guaranteed hygienic conditions.

**Figure 1:**
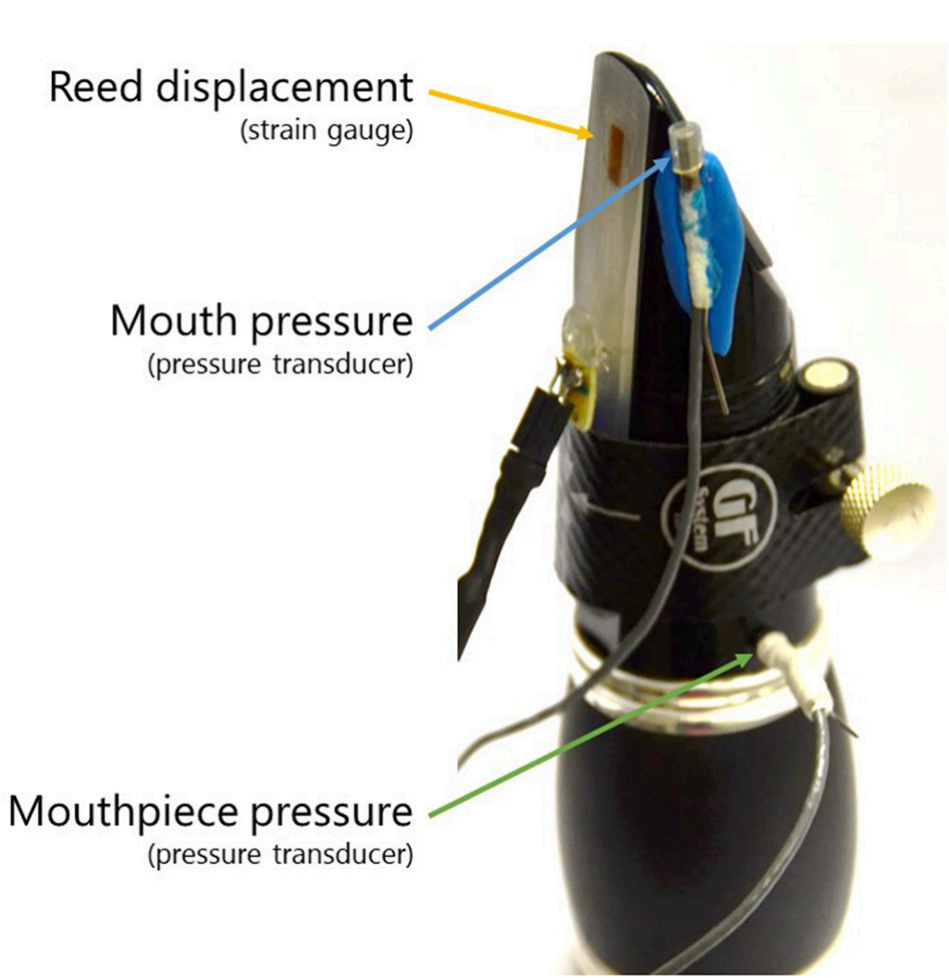
Sensors placed on the clarinet: one strain gauge to measure the reed displacement and two piezo-resistive pressure transducers to measure mouth and mouthpiece pressures.

Acoustic pressures in the mouthpiece and in the player's mouth were recorded via two piezo-resistive pressure transducers (Endevco 8507C-2). One transducer was inserted into the mouthpiece via a side hole at 7.5 cm from the reed tip. The other transducer was attached to the side of the mouthpiece so that it remains inside the player's mouth while playing, as shown in Figure 1. The players were asked to test the comfort of this sensor, with no complaints raised about any change in their playing skills.

The tongue-reed interaction was quantified by considering the bending at the reed surface. To that end, a strain-gauge sensor (2 mm length) was attached to the surface of the reed (German cut, strength 2 ½, by Légère), according to the design presented by Hofmann et al. (2013). Because of the presence of the strain gauge, the reed might increase in stiffness, therefore a soft reed was used. The sensor set-up gives a voltage signal that describes the tensile stress and compression due to reed bending during playing. The strain-gauge signal, the mouth pressure, the mouthpiece pressure, the radiated sound and the metronome signal were simultaneously recorded at 50 kHz using an acquisition platform (NI 9220 with cDAQ-9171, by National Instruments).

### 2.3. Experimental design

**Figure 2:**
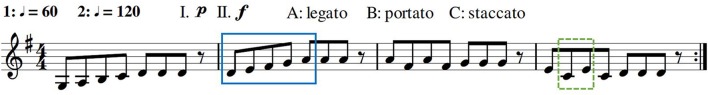
Melody played by the clarinettists, in variations of tempo, dynamic level and articulation. In the blue rectangle, the notes shown in Figures 3, 4; in dashed-green, the note transition shown in Figure 7.

The experiment aims at testing the dependencies of a set of selected parameters on articulation technique, dynamic level and tempo. For this experiment, a melody was designed, consisting of scales, thirds and note repetitions in the chalumeau register of the clarinet (Figure 2). The clarinettists were asked to play the same melody under different performance conditions. Two tempi (60 and 120 bpm with eighth notes) and two dynamic levels (piano and forte) were tested, while considering three articulation techniques (legato, portato, staccato), leading to a 2 × 2 × 3 experimental design. Throughout the entire experiment, the tempo was given by a metronome click and the loudness corresponding to piano and forte was decided by the players. The participants were first provided with the information about the experiment including a consent form. They were informed about the experimental procedure and their right to abort the experiment at any time and to keep their results anonymous. The protocol was approved by the Ethikkomission der Universität für Musik und darstellende Kunst Wien. All subjects gave written informed consent in accordance with the Declaration of Helsinki.

In a warm-up phase, they had about 10 min to get to know the music and familiarise themselves with the instrument and set-up. After that, the experiment started and participants were asked to play the melody according to the 2 × 2 × 3 experimental design, following the tempo of the metronome. At the end, they were asked to answer questions about their instrument practice and playing technique as well as their comfort during the experiment. The whole session took between 40 min and one hour including small breaks when needed.

### 2.4. Data processing

**Figure 3:**
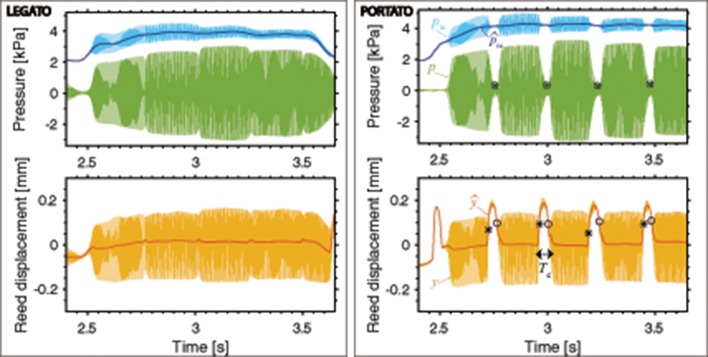
Mouth pressure (*p*_m_, blue), mouthpiece pressure (*p*, green) and reed displacement (*y*, orange), with overlapped low-pass filtered signals (${\hat{p}}_{\text{m}}$ in dark blue, ŷ in red), shown for **legato** and **portato** playing. For portato articulation, on the mouthpiece pressure *p* the landmarks ^*^ and ◦ are found at the instant of minimum pressure amplitude between notes. On the reed signal *y*, the landmarks ^*^ and ◦ are found at the instant of maximum and minimum slope of ŷ to determine the tongue-reed-contact duration *T*_c_. [Player 7: forte, fast playing conditions].

**Figure 4:**
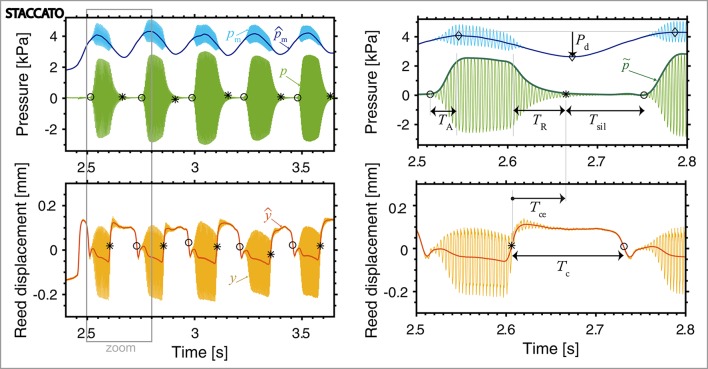
Mouth pressure (*p*_m_, blue), mouthpiece pressure (*p*, green) and reed displacement (*y*, orange), with overlapped low-pass filtered signals (${\hat{p}}_{\text{m}}$ in dark blue, ŷ in red), shown for **staccato** playing. On *p*, the landmarks ◦ and ^*^ indicate the instants of note begin and end (at 5% of the peak amplitude per note). On ŷ, the landmarks ^*^ and ◦ indicate the instants of tongue contact and release (at the instants of maximum and minimum ŷ slope). On the right, a zoomed-in view shows the mouthpiece pressure envelope ($\tilde{p}$, dark green) and the indication of the extracted parameters: *p*_m_ decrease between notes (*P*_d_ obtained between the ◇ landmarks of maximum and minimum mouth pressure), the attack time (*T*_A_) and release time (*T*_R_), the duration of silence-interval between notes (*T*_sil_), the interval from the tongue-contact instant to the sound-end instant (*T*_ce_) and the tongue-reed-contact duration (*T*_c_). [Player 7: forte, fast playing conditions].

Figures 3, 4 show examples of the signals obtained for three different articulation techniques (legato, portato, staccato). For each example, the mouth pressure *p*_m_ (blue) the mouthpiece pressure *p* (green) and the reed displacement *y* (orange) are plotted for 5 notes. A zoomed-in version is given for the staccato playing condition, with indication of the parameters extracted from the signals that we describe next.

#### 2.4.1. Mouth pressure

The pressure inside the mouth of the player *p*_m_ contains a DC and an AC component. The DC corresponds to the pressure provided by the player's lungs (usually called *blowing pressure*, here ${\hat{p}}_{\text{m}}$) and the AC corresponds to the standing waves inside the oral cavity. To obtain the DC mouth pressure independently of the oscillations in the oral cavity (${\hat{p}}_{\text{m}}$, dark-blue signal in Figures 3, 4), a low-pass filter is applied to the original mouth pressure by computing the moving average of the signal with a time-window of approximately the length of one period of the lowest played note (*T*_l_ = 5.6 ms).

The parameters related to the blowing action used in this study are: the mean mouth pressure at each dynamic level and tempo (${\hat{p}}_{\text{m};\text{dynamics},\text{tempo}}$) and the mouth pressure decrease that might occur between notes (*P*_d_ in Figure 4). This mouth pressure decrease is obtained as the difference between the maximum and minimum mouth pressure at every note transition, as indicated with ◇ landmarks in Figure 4.

#### 2.4.2. Mouthpiece pressure

The microphone inserted in the mouthpiece of the clarinet, right before the barrel (Figure 1), measures the sound pressure inside the mouthpiece *p* (green signals in Figures 3, 4). The envelope of the mouthpiece pressure $\tilde{p}$ is computed (low-pass upper-envelope filter, with a window-size of 2*T*_l_) in order to extract parameters to asses the influence of playing techniques on the sound.

Considering the mouthpiece pressure envelope $\tilde{p}$, we characterise the transition between notes in terms of the duration of attack and release transients in portato and staccato articulation (Figures 3, 4). The duration of these transients (*T*_A_ for attack and *T*_R_ for release) is obtained between the instants where the mouthpiece pressure $\tilde{p}$ is at 5% of the peak amplitude per note (landmarks ◦ and ^*^ on the $\tilde{p}$ in Figures 3, 4) and the instants where the amplitude is at 95%. When $\tilde{p}$ does not decrease below 5% of its peak amplitude during the note transition (as is usually the case for portato playing), the minimum amplitude instant between notes is considered as the end of the previous note and the beginning of the next note. For staccato playing, the duration of the silence-interval between notes (*T*_sil_) is computed as the interval between a note end (^*^ in Figure 4) and the next note onset instant (◦ in Figure 4).

#### 2.4.3. Reed displacement

The reed displacement *y* (orange signal in Figures 3, 4) is obtained after calibrating the bending signal captured by a strain gauge attached to the reed (Pàmies-Vilà et al., 2017). The calibration consists of relating the reed-tip displacement to the bending measurement in an artificial blowing set-up where the reed vibration is simultaneously recorded by a high-speed camera and by the strain gauge. When the tongue contacts the reed, the reed vibrations are damped and the reed closes against the mouthpiece lay, resulting in an upwards reed displacement in the recorded signals. In order to isolate the reed motion during tonguing from the reed oscillation, the reed displacement signal is low-pass filtered using a moving-average filter (window of length *T*_l_), ŷ in Figures 3, 4.

The tongue-reed interaction is observed in portato and staccato playing. During tongue-reed contact, the reed is considered to move together with the tongue. The tongue-reed contact instant is determined at the position of maximum slope of the filtered reed signal ŷ (e.g., ^*^ at 2.61 s in Figure 4). The tongue-reed release instant is determined at the position of minimum slope of the filtered reed signal (e.g., ◦ at 2.73 s in Figure 4). By subtracting both time instants, the duration of the tongue-reed contact *T*_c_ is computed.

For one of the players, it was not possible to obtain the tongue-reed contact using this methodology, as he claimed to use a unique tonguing technique consisting of combining tongue and lip modifications to damp the reed vibrations. In this particular technique, the player positions the tongue closer to the lip than to the reed-tip, probably between the strain-gauge and the lip, making it impossible to identify the tongue contact or the tongue release instants in the measured strain-gauge signals. For these reasons, we omit the data of this player.

#### 2.4.4. Statistical analysis

In the next section, the influence of the playing conditions (dynamic level, tempo and articulation technique, which cross over in a 2 × 2 × 3 design) on the different parameters are quantified by an Analysis of Variance or ANOVA (Fisher, 1992). The nomenclature is used as follows: an *n*-way repeated-measures ANOVA analyses the influence of *n* effects or groups (the playing conditions) on a dependent variable (the selected parameters one by one). The notation “repeated-measures” indicates that every participant executed all 12 conditions. The results of the ANOVA are presented as [*F*_(*d, r*)_, *p*, $\eta_{\text{p}}^{2}$], where *F* is the *F*-factor quantifying the influence of an effect on the parameter, *d* are the degrees of freedom for the effect (related to the number of levels of the effect), *r* are the residuals within groups (related to the number of observations, to the number of participants and to the number of levels of the effect), *p* is the *p*-value expressing the significance of the influence and $\eta_{\text{p}}^{2}$ is the partial eta-squared statistic or effect size. A significant effect is considered at a *p*-value of *p* < 0.05 and a high *F*-factor. The effect size $\eta_{\text{p}}^{2}\in \left[0,1\right]$ gives a standardised measure of the magnitude of the observed effect. The reference values are $\eta_{\text{p}}^{2}=0.02$ for a small effect, $\eta_{\text{p}}^{2}=0.13$ for a medium effect and $\eta_{\text{p}}^{2}=0.26$ for a large effect (Bakeman, 2005). A *t*-test is used to compare two conditions and it is reported as [*t*_(*d*)_, *p, r*], where *t* is the *t*-value quantifying the difference between the two conditions, *p* is the *p*-value expressing significance and *r* is the effect size.

## 3. Results

### 3.1. The tonguing technique

The signals in Figures 3, 4 show that, for legato articulation, the note transition happens with a small drop in the sound level at the mouthpiece pressure, whereas in portato, the notes are clearly separated and, in staccato, the notes are separated for a longer time interval. Therefore, the tongue-reed interaction can be only observed for portato and staccato playing techniques, where the tongue damps the vibrations of the reed to stop the sound and separate the notes (Lawson, 1995).

In both portato and staccato playing techniques, we identify the tongue-reed contact in the recorded strain-gauge signals, and obtain the tongue-reed-contact duration (*T*_c_) for every note transition. A Three-way repeated-measures ANOVA on the *T*_c_ to analyse the influence of tempo, dynamics and articulation technique reveals a significant influence of articulation technique [*F*_(1, 9)_ = 22.9, *p* < 0.01, $\eta_{\text{p}}^{2}$ = 0.72], but no significant influence of tempo nor dynamics [*p* > 0.05]. Longer *T*_c_ is used for staccato than for portato, for both slow and fast tempo and by all players. The differences between portato and staccato are further analysed in the following subsections.

#### 3.1.1. Portato articulation

When considering the portato articulation, the mean duration of the tongue-reed contact *T*_c_ across players and playing conditions is 37 ms (standard deviation: 14 ms). A Two-way ANOVA on the *T*_c_ to assess the influence of tempo and dynamics shows that *T*_c_ is independent of both tempo [*F*_(1, 9)_ = 2.55, *p* = 0.14, $\eta_{\text{p}}^{2}$ = 0.22] and dynamic level [*F*_(1, 9)_ = 0.04, *p* = 0.85, $\eta_{\text{p}}^{2}$ = 0.004]. This means that, regarding the duration of the tongue-reed contact, the players present an invariant portato technique regardless of the musical context, confirming the observations made on the saxophone in an earlier study (Hofmann and Goebl, 2014).

#### 3.1.2. Staccato articulation

**Figure 5:**
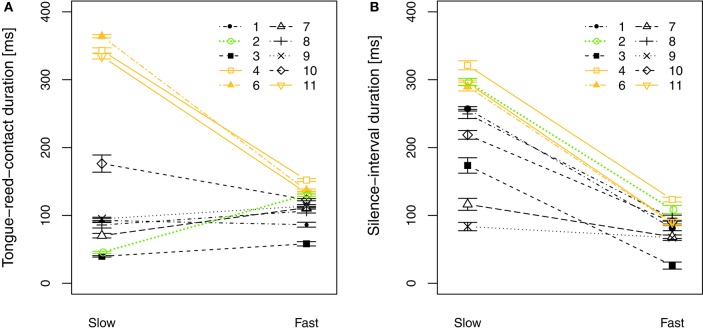
Comparison between tempo conditions in staccato playing for all players (slow: 60 bpm, fast: 120 bpm). **(A)** Tongue-reed-contact duration *T*_c_. **(B)** Silence-interval duration *T*_sil_. Players in orange show a consistency *T*_c_ -to-*T*_sil_ for both slow and fast tempo, whereas players in black and green show a discrepancy *T*_c_ -to-*T*_sil_ in slow tempo. Error bars show the standard error of the mean considering all note transitions in every condition.

In staccato articulation, notes are short and a silence-interval *T*_sil_ appears between notes (Figure 4). When performing a Two-way repeated-measures ANOVA on the tongue-reed-contact duration *T*_c_ regarding tempo and dynamic level in staccato articulation, no significant influence of tempo nor dynamics is found. Figure 5A plots the parameter *T*_c_ for all players comparing slow and fast staccato articulation. It shows that the slow tempo presents a greater variability among players than the fast tempo, suggesting the possibility that different tonguing techniques are used.

Figures 5A,B compare the duration of the tongue-reed-contact *T*_c_ (Figure 5A) to the duration of the silence-interval *T*_sil_ (Figure 5B) during note transitions for all players. It shows that some players present a tongue-reed-contact duration that is as long as the silence-interval duration (4, 6, and 11, highlighted in orange) in both slow and fast tempo. For these players, we can therefore deduce that only the action of the tongue stops the vibration of the reed. Yet all the remaining players (1, 2, 3, 7, 8, 9, 10) present a shorter tongue contact *T*_c_ than the silence-interval *T*_sil_ in the slow tempo. In particular, player 2 (in green) plays a slow staccato with a tongue contact of 46 ms, while the silence-interval takes up to 300 ms. For these players, in slow-staccato playing, the instant of tongue-reed contact does not correspond to the end of the note. This implies that other embouchure modifications than the tongue-reed contact might be used to stop the notes in staccato playing. To further characterise the different techniques that might be used, particularly to stop the notes, a combined analysis considering mouth pressure and tongue-reed interaction is presented in section 3.2.

### 3.2. Blowing to tonguing coordination

In this section, we aim at giving parameter values to characterise the tonguing action and its relationship to the blowing action during tongue-articulated series of notes. The parameter values obtained in this research are given in Table 1, for a hypothetical performance style obtained by considering the average playing technique of all participants.

**Table 1:** Parameter values for portato and staccato articulation techniques [Mean (Standard deviation)], in the order of appearance in the paper: duration of tongue-reed contact *T*_c_, duration of silence-interval between notes *T*_sil_, mean mouth pressure ${\hat{p}}_{\text{m}}$ (*same values for legato), mouth pressure drop during note transition *P*_d_, time interval between the tongue contact and the note end *T*_ce_, mouth pressure at the instant of note end ${\hat{p}}_{\text{m};\text{e}}$, attack time *T*_A_ and release time *T*_R_.

	**Portato**				**Staccato**				
	**Slow**		**Fast**		**Slow**		**Fast**		
	**Piano**	**Forte**	**Piano**	**Forte**	**Piano**	**Forte**	**Piano**	**Forte**	
*T*_c_ [ms]	37 (14)				347 (27)		115 (31)		I
					64 (30)		–		II
*T*_sil_ [ms]	–				302 (43)		84 (37)		I
					196 (91)		–		II
mean ${\hat{p}}_{\text{m}}$ [kPa]^*^	2.7 (0.3)	3.8 (0.6)	2.7 (0.3)	4.1 (0.6)	2.7 (0.3)	3.8 (0.6)	2.7 (0.3)	4.2 (0.6)	I
									II
*P*_d_ [kPa]	–				1.2 (0.4)	2.2 (1.0)	1.1 (0.5)	0.8 (0.5)	I
									II
*T*_ce_ [ms]	–				53 (18)				I
					−137 (64)		–		II
${\hat{p}}_{\text{m};\text{e}}$ [kPa]	–				2.3 (0.6)	3.4 (0.9)	2.3 (0.6)	3.4 (0.9)	I
					1.7 (0.3)		–		II
*T*_A_ [ms]	48 (18)	40 (18)	46 (16)	33 (10)	38 (19)	32 (8)	33 (10)	29 (8)	I
					52 (8)	36 (7)	49 (10)	30 (7)	II
*T*_R_ [ms]	44 (26)				110 (21)		82 (20)		I
					152 (47)				II

*Comparison between portato and staccato, for tempo (slow: 60 bpm, fast: 120 bpm) and dynamic level (piano, forte). On the right, I and II indicate the technique to stop the note in staccato articulation*.

#### 3.2.1. Mouth pressure during performance

The blowing action used during playing is first quantified in terms of the overall mean value of mouth pressure ${\hat{p}}_{\text{m}}$ used during the performance of the melody. A Three-way repeated-measures ANOVA on the mean mouth pressure shows a significant influence of dynamics [*F*_(1, 9)_ = 62.22, *p* < 0.001, $\eta_{\text{p}}^{2}$ = 0.87] as well as a significant influence of tempo [*F*_(1, 9)_ = 33.88, *p* < 0.001, $\eta_{\text{p}}^{2}$ = 0.79] and a dynamics-tempo significant interaction [*F*_(1, 9)_ = 12.30, *p* < 0.05, $\eta_{\text{p}}^{2}$ = 0.58], but the articulation technique does not show a significant influence [*F*_(2, 18)_ = 1.82, *p* = 0.19, $\eta_{\text{p}}^{2}$ = 0.17]. This means that the average mouth pressure used during playing is the same in all three articulation techniques. The influence of dynamics on ${\hat{p}}_{\text{m}}$ is as expected: piano uses lower mouth pressure than forte, therefore the participants adapted the mean ${\hat{p}}_{\text{m}}$ to the required dynamic level. The tempo-dynamics significant interaction on the mean ${\hat{p}}_{\text{m}}$ is given by the fact that in piano ${\hat{p}}_{\text{m}}$ is maintained along tempo conditions (${\hat{p}}_{\text{m};\text{piano}}$ = 2.7 kPa), whereas in forte ${\hat{p}}_{\text{m}}$ increases for faster tempo (${\hat{p}}_{\text{m};\text{forte},\text{slow}}$ = 3.8 kPa, ${\hat{p}}_{\text{m};\text{forte},\text{fast}}$ = 4.2 kPa). This suggests that there is an intention of the player to apply additional character to the music in a fast-forte musical context by providing extra mouth pressure.

A visual inspection of the mouth pressure signals in Figures 3, 4 reveals that the blowing actions differ among playing techniques: for legato and portato (Figure 3) the mouth pressure is maintained at a constant playing level during note transitions, whereas in staccato (Figure 4) a mouth pressure variation appears during note transitions. The relationship between this mouth pressure variation and the tonguing technique is therefore analysed.

#### 3.2.2. Tonguing-blowing interaction in staccato articulation

**Figure 6:**
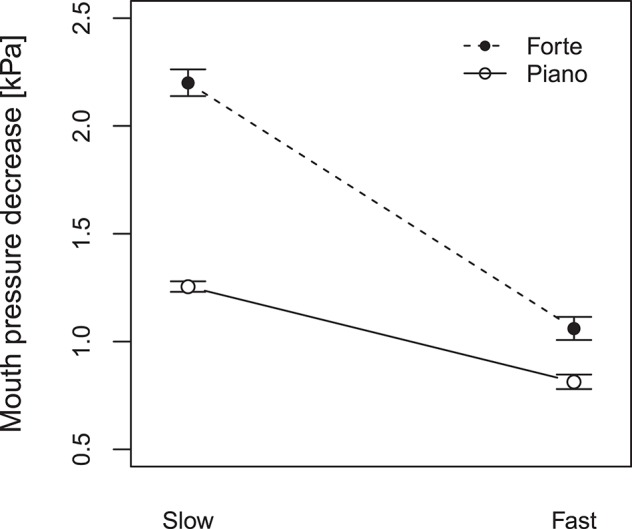
Mouth pressure decrease (*P*_d_) for staccato articulation as a function of tempo (slow: 60 bpm, fast: 120 bpm) and dynamic level (forte, piano). Error bars show the standard error of the mean.

The mouth pressure variation during note transitions present in staccato playing is quantified in terms of the mouth pressure decrease *P*_d_, i.e., the amplitude difference between the upper and the lower values of the ${\hat{p}}_{\text{m}}$ variation (◇ landmarks in Figure 4). This mouth pressure decrease *P*_d_ can be described as a variation of the mouth pressure, which appears between notes in staccato playing and might be used to stop the sound or to create a certain musical expression. A Two-way repeated-measures ANOVA is computed to analyse the effect of tempo and dynamics on *P*_d_ considering all note transitions in staccato articulation. As shown in Figure 6, significantly larger *P*_d_ is observed when playing forte than when playing piano [Dynamics: *F*_(1, 9)_ = 12.1, *p* < 0.05, $\eta_{\text{p}}^{2}$ = 0.57]; and significantly larger *P*_d_ appears in slow than in fast tempo [Tempo: *F*_(1, 9)_ = 17.4, *p* < 0.05, $\eta_{\text{p}}^{2}$ = 0.66]. In fast tempo, we observe that the dynamic level influence is smaller than in slow tempo, giving a significant interaction between the two factors [*F*_(1, 9)_ = 6.33, *p* < 0.05, $\eta_{\text{p}}^{2}$ = 0.41]. The widest *P*_d_ appears for playing conditions with slow tempo and forte dynamics, with values up to 2.2 kPa. For fast playing, a variation of about 1 kPa is present (e.g., Figure 4). It is therefore postulated that in the fast tempo there is not enough time between notes for a pressure variation to occur at the same range as in the slow tempo.

**Figure 7:**
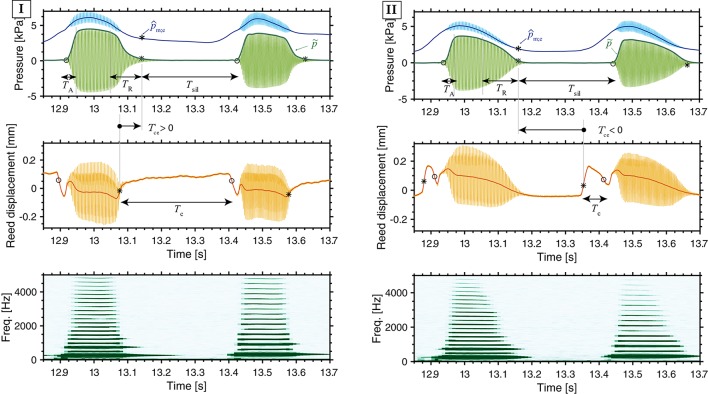
Mouth pressure (blue), mouthpiece pressure (green), reed displacement (orange) and spectrogram of the mouthpiece pressure, showing two examples of staccato articulation. I: The tongue action stops the sound (*T*_ce_ > 0). II: A decrease in the mouth pressure stops the sound and the tongue action comes after the sound has ended (*T*_ce_ < 0). At the instant of note end, the mouth pressure is indicated as ${\hat{p}}_{\text{m};\text{e}}$. From the mouthpiece pressure envelope $\tilde{p}$, the attack time *T*_A_ and release time *T*_R_ and the silence-interval *T*_sil_ are obtained. On the reed displacement, the tongue-reed-contact duration is noted as *T*_c_. [Players 11 and 2, staccato, forte, slow playing conditions].

We have reported in section 3.1.2 that some players do not use the tongue contact to stop the reed vibrations in staccato articulation (particularly for slow tempo conditions), and we have then shown that a mouth pressure variation appears between notes when playing staccato. These observations suggest that players might combine tonguing and blowing actions when playing staccato. Next we aim at analysing how the two actions interact to perform a certain sound effect when playing staccato. For this analysis we consider two parameters: the time interval between the instant when the tongue contacts the reed and the instant of note end (*T*_ce_), and the mouth pressure value at the instant of note end (${\hat{p}}_{\text{m};\text{e}}$). The time landmarks related to these parameters are marked with ^*^ in Figure 7. As we are interested in studying the characteristics of note transitions, the last note release of every musical phrase is omitted.

Figure 7 shows two different ways of stopping a note in staccato playing. In strategy I, the note transition presents longer duration of tongue-reed contact *T*_c_ than in strategy II. In I, the tongue action stops the reed vibration resulting in a positive *T*_ce_, whereas in II, the tongue action comes after the sound has stopped (*T*_ce_ < 0). In strategy I, the value of ${\hat{p}}_{\text{m};\text{e}}$ does not directly affect the instant of sound end, as the player chooses when to stop the sound with the action of the tongue. This means that the player can maintain a high pressure in the mouth or use a certain mouth pressure modulation *P*_d_ between notes. However, in strategy II, it is required to decrease the mouth pressure so as to achieve a value of ${\hat{p}}_{\text{m};\text{e}}$ low enough to stop the sound between notes.

**Figure 8:**
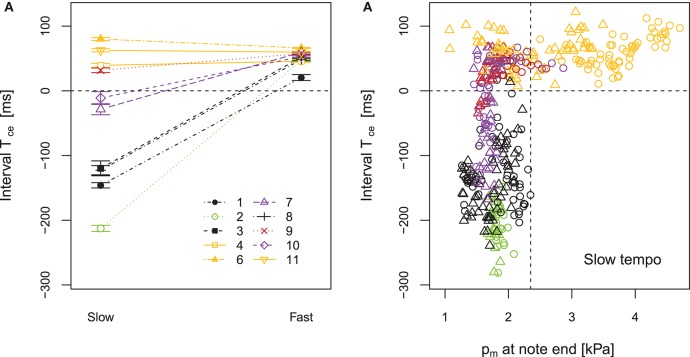
Comparison among participants in staccato playing. **(A)** Tongue-contact to note-end interval *T*_ce_ as a function of tempo (slow: 60 bpm, fast: 120 bpm). **(B)** *T*_ce_ vs. ${\hat{p}}_{\text{m};\text{e}}$ (mouth pressure at the instant of note-end) scatter plot of all note transitions in slow-staccato playing. ◦ corresponds to forte and △ corresponds to piano dynamic level. Error bars show the standard error of the mean considering all note transitions in every condition.

Considering players individually, Figure 8A plots the parameter *T*_ce_ comparing slow and fast tempo and Figure 8B plots the comparison between the parameters *T*_ce_ and ${\hat{p}}_{\text{m};\text{e}}$ in slow staccato playing. These plots show that players 4, 6, 9, and 11 always present a positive *T*_ce_ in slow tempo, meaning that they stop the sound with the action of the tongue (Figure 8, strategy I). This includes the players that showed the longest tongue-reed-contact duration *T*_c_ in Figure 5 (highlighted in orange). Players 1, 2, 3, and 8 present the greatest contrast between slow and fast tempo techniques, showing a very low *T*_ce_ in slow tempo. These players are consistent in stopping the notes by decreasing the mouth pressure and only using the tongue-reed contact right before the beginning of the next tone (strategy II). Players 7 and 10 (highlighted in purple) are not consistent in using one strategy, but rather use a combination of I and II, as they present positive and negative *T*_ce_ values. For fast tempo, all players present a positive *T*_ce_ (strategy I).

The comparison *T*_ce_ vs. ${\hat{p}}_{\text{m};\text{e}}$ in slow-staccato playing (Figure 8B) allows to distinguish the two strategies according to the value of mouth pressure at the end of each note (${\hat{p}}_{\text{m};\text{e}}$). The players presenting positive *T*_ce_ stop the notes at high mouth pressure ${\hat{p}}_{\text{m};\text{e}}\in \left[1,5\right]$ kPa (players highlighted in orange), whereas the players with negative *T*_ce_ drop the mouth pressure down to ${\hat{p}}_{\text{m};\text{e}}<2.3$ kPa (vertical dashed line in Figure 8B). Regarding the influence of dynamics (◦ for forte and △ for piano), Figure 8B shows that in strategy I (*T*_ce_>0), the value of ${\hat{p}}_{\text{m};\text{e}}$ is related to the dynamic level (${\hat{p}}_{\text{m};\text{e};\text{piano}}<{\hat{p}}_{\text{m};\text{e};\text{forte}}$). But in strategy II (*T*_ce_ < 0), ${\hat{p}}_{\text{m};\text{e}}$ is not influenced by dynamics.

### 3.3. Influence of the playing technique on the sound

In order to assess to which extend the tonguing and blowing actions affect the produced sound, we analyse the envelope and the spectrogram of the mouthpiece pressure ($\tilde{p}$ at the top and spectrogram at the bottom of Figure 7). The pressure in the mouthpiece is the parameter that relates the most to the external sound and has the advantage of not being modified by the room reflections. The mouthpiece pressure is recorded in the same experimental conditions for all participants, whereas the external sound is affected by the movement of the instrument.

To confirm the observations made on the blowing technique, a *t*-test compares the mean envelope mouthpiece pressure ($\tilde{p}$) between piano and forte conditions. The *t*-test shows that participants used significantly different dynamics in forte and in piano playing conditions [*t*_(59)_ = 19.21, *p* < 0.001, *r* = 0.93]. All players showed louder dynamics in forte (mean $\tilde{p}$ of 2.6 kPa, SD 0.4 kPa) than in piano (mean $\tilde{p}$ of 1.7 kPa, SD 0.2 kPa).

**Figure 9:**
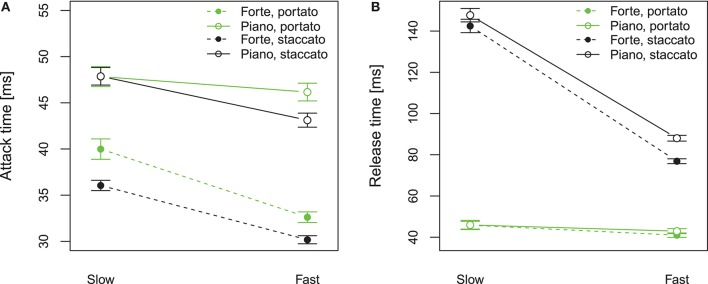
Attack time **(A)** and release time **(B)** measured at the mouthpiece pressure (*T*_A_ and *T*_R_), in portato (green) and staccato (black) playing, comparing tempo (slow: 60 bpm, fast: 120 bpm) and dynamics (forte, piano). Error bars show the standard error of the mean considering all note transitions.

As the articulation technique is strongly linked to the attack and release transients of notes (Lawson, 1995), we first consider the duration of these transients (*T*_A_ and *T*_R_, Figure 7) and we aim at detailing their relationship to the playing parameters described in the previous sections. Figure 9 plots the attack and release times comparing tempo and dynamics, for portato (green) and staccato (black).

Regarding the portato articulation, a Two-way ANOVA on the attack time *T*_A_ shows a significant influence of dynamics [*F*_(1, 9)_ = 40.12, *p* < 0.001, $\eta_{\text{p}}^{2}$ = 0.82]. As shown in green in Figure 9A, forte dynamic level shows shorter attack time than piano. The significant interaction between tempo and dynamics [*F*_(1, 9)_ = 9.042, *p* < 0.05, $\eta_{\text{p}}^{2}$ = 0.50] is given because the forte configuration shows a significant influence of tempo (fast tempo shows shorter attack time than slow tempo) but the piano configuration does not. The same analysis for the release time *T*_R_ (green in Figure 9B), shows no significant effect of tempo [*F*_(1, 9)_ = 0.4, *p* = 0.53, $\eta_{\text{p}}^{2}$ = 0.04] and dynamics [*F*_(1, 9)_ = 0.2, *p* = 0.67, $\eta_{\text{p}}^{2}$ = 0.02] and no interaction. This means that the attack time in portato is related to the playing conditions (tempo, dynamics) but the release time is independent of both. These results are linked to the previously-reported tonguing and blowing characteristics, as the attack transient behaves according to the mouth pressure (shorter attacks are given at higher mouth pressure) and the release transient behaves according to the tongue-reed-contact duration, which appears invariant among all playing conditions (see *T*_A_ and *T*_R_ in Table 1).

In regard to the staccato articulation (black in Figure 9), a Two-way ANOVA shows that the attack time *T*_A_ is affected by tempo [*F*_(1, 9)_ = 17.36, *p* < 0.05, $\eta_{\text{p}}^{2}$ = 0.65] and by dynamics [*F*_(1, 9)_ = 33.71, *p* < 0.001, $\eta_{\text{p}}^{2}$ = 0.79], as fast tempo and forte dynamics present shorter attack time than slow tempo and piano dynamics (Figure 9A). The same analysis for the release time *T*_R_ (black in Figure 9B), reveals a strong influence of tempo [*F*_(1, 9)_ = 26.33, *p* < 0.001, $\eta_{\text{p}}^{2}$ = 0.74], as slow tempo presents much longer release time than fast tempo, but no significant influence of dynamics [*F*_(1, 9)_ = 3.25, *p* = 0.10, $\eta_{\text{p}}^{2}$ = 0.26]. The release time is highly affected by the tempo condition, suggesting a relationship to the techniques to stop the reed vibration in slow staccato playing presented in Figure 7.

In view of these results and previously-mentioned observations on the tonguing technique, a closer look into playing techniques of individual players is required. A visual inspection of the envelope and spectrogram of the mouthpiece pressure signal (in green in Figure 7) shows that big differences appear in the release transient between the techniques I and II. The envelope of the sound in the mouthpiece drops exponentially when stopping the reed vibrations with the tongue (I in Figure 7) but approximately linearly when applying a mouth-pressure decrease (II). This translates into shorter release times in strategy I than in II. Regarding the spectral content (bottom of Figure 7), in strategy I, at the instant of tongue-reed contact the upper harmonics are simultaneously damped and only the low odd harmonics resonate in the tube until the sound completely stops; whereas in strategy II, the harmonics are damped progressively from higher to lower frequencies during the mouth pressure decrease.

**Figure 10:**
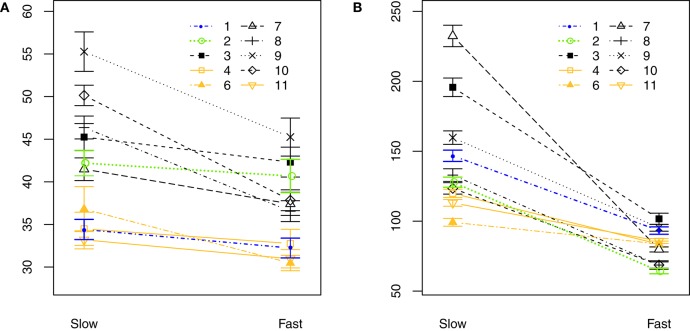
Attack **(A)** and release **(B)** times measured at the mouthpiece pressure (*T*_A_ and *T*_R_ in Figure 7), in **staccato** playing, comparing tempo (slow: 60 bpm, fast: 120 bpm) for all players. Error bars show the standard error considering all note transitions in every condition.

Figure 10 shows the attack and release time for all players in staccato articulation. Both attack and release times are influenced by tempo, as all players present shorter transients in fast tempo than in slow tempo. Regarding the attack time (Figure 10A), all players present a difference between the slow and the fast tempo condition of about 5–10 s, and the values are similarly spread in slow and fast. In contrast, the difference between slow and fast tempo in the release time (Figure 10B) is highly player-dependent, showing spread values for slow tempo and concentrated values for fast tempo.

The individualities shown on the release time (Figure 10B) correspond to the observations made for the blowing-to-tonguing coordination (section 3): in slow tempo the players using the tongue to stop the sound (technique I; players 4, 6, 11, in orange) present shorter release times than players decreasing the mouth pressure to stop the sound (technique II), yet all players present similar release times for fast tempo. This confirms that some players use technique I for all playing conditions whereas other players adapt their playing technique according to the musical context.

The fact that fast playing is more homogeneous among players than slow playing can be explained considering that in fast tempo the time interval between notes is too short to let the mouth pressure decrease enough to stop the sound between notes, thus requiring the action of the tongue. However, in the slow tempo, no consensus exists among players whether to play the staccato release with the blowing action or with the tonguing action when there is enough time between notes to choose either option. Using one strategy or the other is not correlated to the years of experience (professional players or students) and also not to the hours of practice per week. Note that the number of professional participants is small and does not allow conclusions to be drawn regarding the level of expertise. It is the player's choice whether to stop the notes with the tongue or with the blowing action, hence creating a sharp or smooth release.

A noteworthy observation is that the manner the note is stopped (technique I or II) influences not only the release time but also the attack time (Figure 10A). The players using the strategy I (players 4, 6, 11 in orange) show a shorter attack transient than the players using strategy II. An exception of this influence is player 1 (in blue in Figure 10), who uses strategy II, therefore creates long release times, but still obtains a short attack time. Moreover, the influence of the tonguing technique on the attack time is not only present in slow tempo but also in fast tempo, where all players use the tongue to stop the notes (strategy I). This suggests that players using strategy I in all tempo conditions are able to always create short transients, and provide consistency among tempo conditions. Whereas the players that choose to stop the notes with a mouth pressure decrease in slow tempo cannot maintain the same strategy in fast tempo, thus introducing variability among tempo conditions, which can be seen as a desired effect or limitation of their technique.

These results show that each note played in portato and staccato articulation presents an attack and a release transient that are linked to the tonguing technique and mouth pressure involved in generating it. Portato articulation presents attack and release times with values of the same order of magnitude, resulting in a symmetrical envelope in the mouthpiece pressure during note transitions (right of Figure 3). In staccato, the behaviour of the attacks is similar to that of portato (Figure 9A) but the release times are always longer. Both in portato and staccato, the attack transients (Table 1 *T*_A_) are more dependent on the player's mouth pressure than the release transients (Table 1 *T*_R_), as the attack transient is faster when the dynamic level is louder. The release transient instead, does not depend on dynamics but depends on the technique used to stop the reed vibrations (I and II in Table 1).

## 4. Discussion

The technique to perform clarinet articulation is controlled by different actions in the player's mouth. The characteristics of articulation have been studied by extracting and analysing a set of parameters obtained when playing a series of articulated notes. These parameters are related to the tonguing action, the blowing action and the sound. They have been used to analyse the influence of tempo and dynamics on the articulation technique. Even though differences between participants demonstrate that articulation can be highly player-dependent, we report which actions lead to sound qualities that characterise the articulation techniques.

The results show that legato and portato articulation present homogeneous characteristics among players but staccato shows more individual variations. The mouth pressure is mainly adapted to the dynamic level and the tonguing action is adapted to the articulation technique. In portato the tonguing technique was the same in all of the playing conditions, consisting of stopping the reed vibrations with a short tongue stroke while maintaining the blowing action between notes. This confirms the results of previous studies on isolated notes (Li et al., 2016) and on note repetitions (Hofmann and Goebl, 2014). The novelty of the current study is to combine tonguing measurements with pressure measurements to assess the performance of a musical phrase in variation of articulation technique, dynamic level and tempo.

The articulation techniques, tempo and dynamics were chosen to provide contrast (piano-forte, slow-fast) and explore different playing techniques. However, a limitation of this study is that such controlled experimental conditions can only reflect an excerpt of performance strategies and do not cover all the phenomena that might be used in a more musical context. Particularly, the melody used had a regular rhythm played in one register of the clarinet. In changes of register or in a more complex musical phrase, the playing technique can differ from the observations made in this study. Regarding the dynamic levels, the instructions during the experiment were given by the musical terms “piano” and “forte.” It was nonetheless verified that all players used significantly different dynamics for both experimental conditions.

The staccato articulation was highly variable between participants. We observed that there was a decrease in mouth pressure between notes. This decrease is used by some players to stop the reed vibrations in slow staccato articulation. We classified the observed playing technique into two main strategies on the basis of how reed vibration was terminated between notes. When the tongue stops the note, the release transient is short and the harmonic content in the mouthpiece pressure is damped abruptly; when the mouth pressure stops the note, the release transient is longer and the harmonics are damped successively. Moreover, the implications of using one or the other technique are not only on the release transients but also on the attack transients. When using the tongue to stop the notes, the attack transients are shorter than when using the mouth pressure. These findings show that the player's actions involved during note transitions have a major effect on the transients of notes. The faster the tempo, the more it is necessary to stop the notes with the tongue, as a decrease in the blowing pressure would require more time than the time interval between notes. It is for this reason that using the blowing pressure in slow tempo makes the staccato technique differ among tempi (smooth in slow and hard in fast tempo). This can be a desired effect or may be a consequence of the technique used even if unwanted. Considering that staccato is characterised by having short notes and fast transients, it can be asserted that using the tongue to stop the reed vibrations allows the player to obtain these characteristics rather than using mouth pressure variations.

The current empirical study broadens the knowledge on articulation in single-reed woodwinds and provides physically meaningful values to characterise the parameters related to articulation on the clarinet (Table 1). A possible application of these findings is to inform physical models of woodwind instruments. Such models could consider the obtained values as a reference for the simulation of articulatory player actions (Chatziioannou and Hofmann, 2015). In a pedagogical context, the current methodology of using sensor-equipped instruments to monitor player actions can be seen as a tool from which clarinet teachers and students may benefit. Such methods give insights about the hidden actions taking place inside the player's mouth, which are usually only described in words throughout the teaching processes. It allows to compare playing techniques (namely the playing technique of the teacher compared to that of the student) and distinguishes the main parameters controlling articulation: how long does the tongue stay on the reed and how does the mouth pressure evolve during articulation.

## Author contributions

MP-V, AH, and VC: Design of the experiment. MP: Accomplishment of the experiment, data processing and analysis, and writing of report. AH and VC: Research supervision.

### Conflict of interest statement

The authors declare that the research was conducted in the absence of any commercial or financial relationships that could be construed as a potential conflict of interest.
